# Long-Term Dietary Folate Deficiency Accelerates Progressive Hearing Loss on CBA/Ca Mice

**DOI:** 10.3389/fnagi.2016.00209

**Published:** 2016-08-31

**Authors:** Raquel Martínez-Vega, Silvia Murillo-Cuesta, Teresa Partearroyo, Gregorio Varela-Moreiras, Isabel Varela-Nieto, María A. Pajares

**Affiliations:** ^1^Instituto de Investigaciones Biomédicas Alberto Sols (CSIC-UAM)Madrid, Spain; ^2^Unidad 761, Centro de Investigación Biomédica en Red de Enfermedades Raras (CIBERER), Instituto de Salud Carlos IIIMadrid, Spain; ^3^Instituto de Investigación Hospital Universitario La Paz (IdiPAZ)Madrid, Spain; ^4^Departamento de Ciencias Farmacéuticas y de la Salud, Facultad de Farmacia, Universidad CEU San PabloMadrid, Spain

**Keywords:** vitamin deficiency, hearing impairment, homocysteine, cochlea, dietary intervention, hyperhomocysteinemia, folic acid

## Abstract

Dietary folic acid deficiency induced early hearing loss in C57BL/6J mice after 2-months, corroborates the epidemiological association previously described between vitamin deficiency and this sensory impairment. However, this strain is prone to early hearing loss, and hence we decided to analyze whether the effects exerted by folate deprivation follow the same pattern in a mouse strain such as CBA/Ca, which is resistant to hearing impairment. Here, we show results of a long-term study on hearing carried out on CBA/Ca mice subjected to dietary folate deprivation. Systemic changes included decreased serum folate levels, hyperhomocysteinemia and signs of anemia in the group fed with folate-deficient (FD) diet. Initial signs of hearing loss were detected in this strain after 8-months of vitamin deficiency, and correlated with histological damage in the cochleae. In conclusion, the data presented reinforce the importance of adequate folic acid levels for the auditory system and suggest that the impact of dietary deficiencies may depend on the genetic background.

## Introduction

Hearing loss is a sensory impairment caused by genetic and environmental factors (Dror and Avraham, 2009; Roth et al., 2011), whose incidence is increasing according to reports of the World Health Organization. Factors such as noise and ototoxic drugs are well-known effectors of this impairment, but more recently several epidemiological studies have shown the association between the nutritional status and hearing loss. Precisely, inadequate levels of folic acid have been correlated with hearing loss in the presence of reduced vitamin B_12_ concentrations or hyperhomocysteinemia (Houston et al., 1999; Cadoni et al., 2004; Lasisi et al., 2010). Additional reports have explored the putative association of mutations in genes of the folate cycle and the impact of hearing loss, their results being somewhat inconsistent (Durga et al., 2006; Uchida et al., 2011). In contrast, two animal studies have demonstrated the link between homocysteine (Hcy) metabolism and hearing loss (Cohen-Salmon et al., 2007; Martinez-Vega et al., 2015a) and the effects of a folate-deficient (FD) diet in the auditory system (Martinez-Vega et al., 2015a). Thus, remarkable increases in auditory brainstem response (ABR) thresholds were detected in C57BL/6J mice after 2-months on a FD diet (Martinez-Vega et al., 2015a). These changes correlated with the presence of severe histological damage in the cochlea and with important alterations in cochlear metabolism. Folate deficiency induced a decrease in Hcy remethylation and its flux through trans-sulfuration, together with an increase in adenosine elimination, as deduced from the important changes observed in the enzymes involved. Altogether these changes led to cochlear accumulation of Hcy, resulting in enhanced protein homocysteinylation, and in redox stress in FD cochleae (Martinez-Vega et al., 2015a). C57BL/6J is a strain known to develop premature hearing loss, a phenotypic trait that was accelerated in FD mice. Therefore, we set up a new experiment using the CBA/Ca mouse strain, less prone to hearing loss, to further evaluate the impact of folate deficiency in the development of this impairment.

## Materials and Methods

### Mouse Handling and Experimental Design

Two month-old CBA/Ca female mice were purchased from Harlan Interfauna Ibérica S.A. and housed under standard conditions. Mice were randomly divided into two experimental groups (*n* = 10 each) that were fed the A04/A04C/R04 diet (Panlab/SAFE) containing standard folate levels (normal folate 2 mg/kg; NF) or a FD diet (folic acid ≤0.1–0.2 mg/kg, Harlan Tecklad TD.95247) *ad libitum* for 8 months. Weight gain was measured weekly for both dietary regimes. All experiments were approved by the CSIC Bioethics Committee and carried out in full accordance with the guidelines of the European Community (2010/63/EU) and the Spanish regulations (RD 53/2013).

### Blood Analysis and Histology

Blood samples were collected through the external maxillary vein (2-months of age; 2M) or by cardiac puncture after CO_2_ asphyxiation (10-months of age; 10M), and directly placed in either regular or heparin coated tubes (Laboratorios Farmacéuticos Rovi). Isolation of serum and plasma fractions was performed after centrifugation at 2500× g for 10 min. The hematological analysis was performed with Abacus Junior Vet 5 automatic equipment (Diatron). Fresh blood (2/mice) extensions were prepared for Wright staining (Reagan et al., 2011).

For histological analysis mice were injected a pentobarbital overdose and perfused with PBS/paraformaldehyde as previously described (Camarero et al., 2001; Sanchez-Calderon et al., 2010), before tissue extraction (cochlea and femur; Rodriguez-de la Rosa et al., 2014). Decalcified samples were dehydrated and embedded in paraffin (Panreac Química), as previously described (Aburto et al., 2012). Cochlear and bone marrow cytoarchitecture was evaluated using representative paraffin sections (7 μm thick) and Nissl or hematoxylin-eosin staining (Reagan et al., 2011).

### Hearing Assessment

Mice were anesthetized with a mixture of ketamine (100 mg/kg; Imalgene 1000, Merial) and xylazine (10 mg/kg; Rompun 2%, Bayer) for ABR analysis using a Tucker Davis Technologies workstation. The electrical responses to broadband click and 8, 16, 20, 28 and 40 kHz pure tone stimuli, with an intensity range 90–20 dB SPL in 5–10 dB steps, were recorded as previously reported (Cediel et al., 2006; Murillo-Cuesta et al., 2012). The electrical responses were amplified and averaged to determine hearing thresholds for each stimulus. Peak and interpeak latencies were analyzed at 15–20 dB SPL above hearing threshold after click stimulation. Recording of distortion product otoacoustic emissions (DPOAEs) was performed after stimulation with f1 and f2 primary tones, with a ratio f2/f1 = 1.2 using a TDT equipment, as described previously (Martinez-Vega et al., 2015b). Primary tones for 8, 10, 14, 18 and 22 kHz frequencies were tested.

### Metabolite Determinations

Total Hcy (tHcy) was derivatized using the Reagent kit for HPLC analysis of Hcy in plasma/serum (Chromsystems Instruments and Chemicals GmbH, Munich, Germany). The resulting samples (50 μl) were then injected into the HPLC column and fluorescence measured at 515 nm upon excitation at 385 nm.

Total serum folate was determined using the microbiological method developed by Horne and Patterson (1988) with Tamura’s modifications (Tamura, 1990).

### Statistical Analysis

Statistical analysis was carried out between NF and FD groups at the specified time points using the Student’s *t*-test for unpaired samples was performed with SPSS v 19.0 software package (SPSS, Chicago, IL, USA). No statistical evaluation was performed within each dietary group during the whole experiment.

## Results

**Figure 1:**
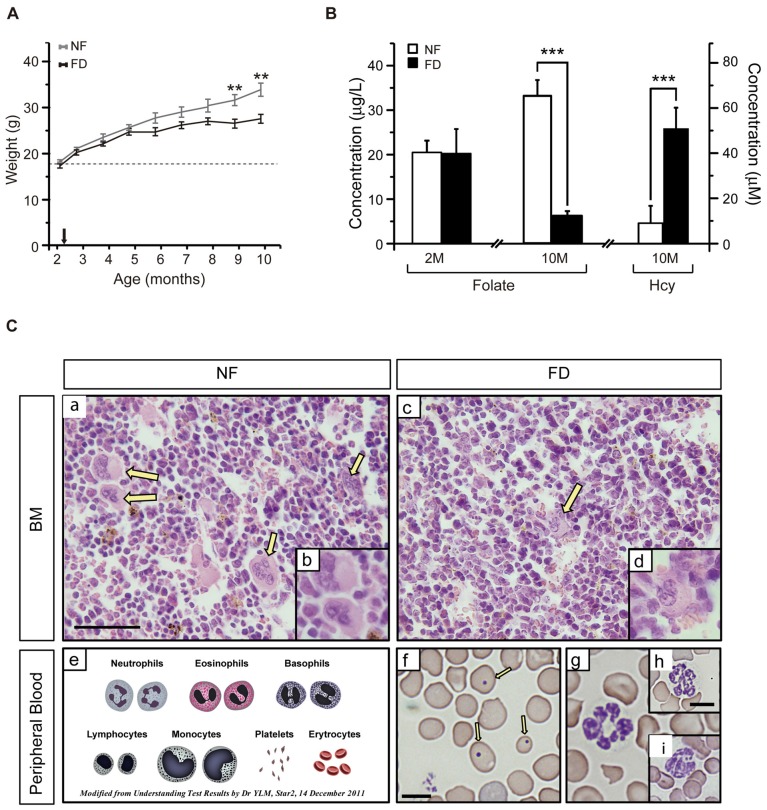
**(A)** Weight gain along the study. The size of each group is specified in Table 2. Several mice from the folate-deficient (FD) group were sacrificed due to their condition, and hence the group size was reduced (*n* = 5) from 7 months of age onward. **(B)** Serum folate concentrations were measured at 2-months of age (2M) in the normal folate (NF; *n* = 9) and FD (*n* = 5) groups. At 10-months (10M) of age serum folate and homocysteine (Hcy) concentrations were determined in the NF (*n* = 9) and FD groups (*n* = 5). **(C)** Hematoxylin-eosin staining of bone marrow **(a–d)** and Wright staining of peripheral blood **(f–i)** at 10M (*n* = 5 for both dietary groups). Megakaryocytes (yellow arrows), scheme of normal erythrocytes and white series **(e)**, anisocytosis and Howell-Jolly bodies (**f**, arrows). Bar scale 50 μm **(a,c)**; 25 μm **(b,d)**; 10 μm (remaining panels). Statistical analysis was performed between NF and FD groups at each of the specified time points; no evaluation within the same dietary groups was performed. **p < 0.01; ***p < 0.001.

The CBA/Ca mouse strain, known to present with late-onset of hearing loss (18-months onward; Li and Borg, 1991; Zheng et al., 1999), was chosen to evaluate the impact of a FD diet during a long-term study carried out for 8 months. No statistical differences in the daily food intake were found between NF and FD groups during the experiment. However, animals in the FD group gained less weight than those on the NF diet, this difference becoming significant at 9–10 months of age (Figure 1A). Nevertheless, both daily ingestion and weight gain remained above normal values for this strain^1^.

Additional effects of the dietary treatment were analyzed in blood samples obtained at the start and the endpoint of the study (Figure 1B). No differences in serum folate levels between groups were detected at the beginning of the experiment (NF-2M vs. FD-2M), whereas a five-fold decrease was measured in the FD-10M group. This decrease correlated with a seven-fold increase in tHcy levels in FD-10M mice (Figure 1B), as expected for the role of folate in Hcy remethylation. Altogether, these systemic metabolic changes confirmed the efficacy of the dietary treatment.

FD-10M mice also presented decreased hemoglobin levels, packed cell volume and hematocrit values, lower red and white blood cell counts and reduced lymphocyte percentage, together with increased mean corpuscular volume, mean corpuscular hemoglobin and an enhanced degree of anisocytosis (Table 1). Their bone marrow showed a decrease in megakaryocyte number, which also had lower differentiation and volume (Figure 1C). Along with these alterations an increased number of Howell-Jolly bodies, hypersegmented polymorphonuclear neutrophils and strong anisocytosis were observed in peripheral blood extensions of FD-10M mice (Figure 1C). Altogether, these changes indicated signs of megaloblastic anemia that jointly with the presence of signs of stress in the FD-10M mice, led to the interruption of our study at 10M, despite the late-onset of HL described for these mice.

**Table 1:** **Blood parameters of CBA/Ca mice**.

	10 months of age	
	NF^1^ (*n* = 5)	FD^1^ (*n* = 5)
White blood cells (10^9^/L)	6.36 ± 0.23	3.10 ± 0.52***
Red blood cells (10^12^/L)	9.53 ± 0.12	6.61 ± 0.30***
Hemoglobin (g/dL)	14.08 ± 0.34	11.38 ± 0.51**
Hematocrit (%)	46.88 ± 0.80	38.43 ± 1.32**
Mean corpuscular volume (fL)	49.50 ± 0.29	58.25 ± 0.75***
Mean corpuscular hemoglobin concentration (g/dL)	30.03 ± 0.45	29.63 ± 0.61
Mean corpuscular hemoglobin (pg)	14.78 ± 0.25	17.28 ± 0.34***
Anisocytosis (%)	17.28 ± 0.28	20.58 ± 0.54**
Granulocytes (%)	22.06 ± 1.01	29.03 ± 2.35
Lymphocytes (%)	75.65 ± 1.36	52.53 ± 7.29*
Mid-range cells (%)	4.60 ± 1.15	6.43 ± 1.44

Blood parameters were measured at the end of the study, when the mice were 10 months of age, and had been fed the normal (NF) or folate deficient (FD) diets for 8 months. Statistical analysis was performed between NF and FD groups. ^1^mean ± SEM; *p < 0.05; **p < 0.01; ***p < 0.001.

**Figure 2:**
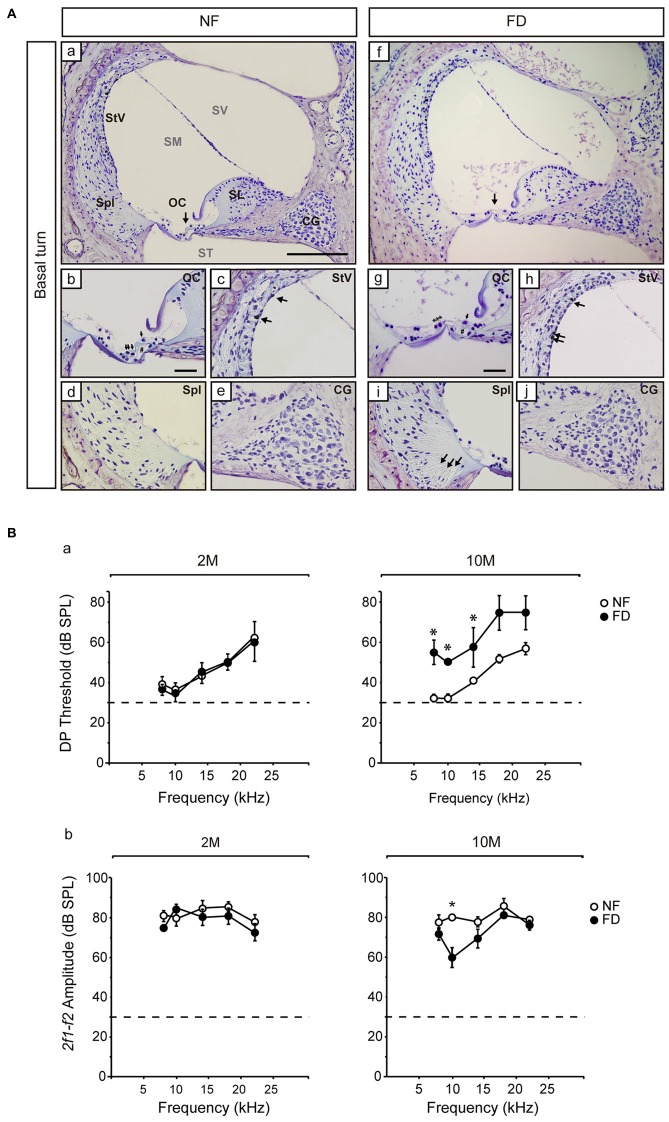
**Representative histological images of the Nissl-stained basal regions and distortion product otoacoustic emissions (DPOAE) results. (A)** General views of the cochlear basal turn **(a,f)** for NF (*n* = 3) and FD mice (*n* = 3) 10-months old. Detailed views of the organ of Corti (OC; **b,g**), where collapse of the inner and outer pillar cells is observed in both dietary groups (#) and the presence of outer hair cells in the NF (arrows) and their absence in the FD group (***) are indicated. Additional subpanels show detailed views of the *stria vascularis* (StV; **c,h**) with melanin granules in both dietary groups (arrows); spiral ligament (Spl; **d,i**) and cochlear ganglion (CG; **e,j**). Bar scale: 150 μm **(a,f)**; 25 μm (remaining panels). **(B)** DPOAE analysis at 2- (2M; NF *n* = 9 and FD *n* = 10) and 10-months (10M; NF *n* = 9 and FD *n* = 5) of age; DP thresholds **(a)** and *2f1-f2* amplitudes **(b)** are shown (mean ± SEM). *p < 0.05.

The auditory function was evaluated every month in both dietary groups. No significant changes in ABR threshold, latencies, or amplitudes were evident between NF and FD mice along the whole study (Table 2). Both NF-10M and FD-10M groups showed a decreased amplitude of wave I, which correlated with a slight increase in hearing thresholds. Histological analysis revealed damage in the organ of Corti (OC) at the low basal turn of the cochlea, together with a slight loss in type IV fibrocytes at the spiral ligament (Spl) and accumulation of melanin granules in the *stria vascularis* (StV; Figure 2A). Altogether, these alterations are indicative of the initial phases of hearing loss in both dietary groups, but seem more severe in cochleae of FD mice in which the absence of outer hair cells is noticed (Figure 2Ag).

**Table 2:** **Auditory brainstem response (ABR) results at three points of the study**.

		Age					
		2 months		7 months		10 months	
		NF (*n* = 9)	FD (*n* = 10)	NF (*n* = 9)	FD (*n* = 9)	NF (*n* = 9)	FD (*n* = 5)
ABR^1^ threshold (dB SPL)	Click	16 ± 1	17 ± 1	16 ± 1	18 ± 1	24 ± 1	24 ± 3
	8 kHz	22 ± 1	25 ± 0	23 ± 1	23 ± 1	44 ± 4	43 ± 1
	16 kHz	21 ± 1	25 ± 2	24 ± 1	25 ± 0	43 ± 5	43 ± 3
	20 kHz	21 ± 1	25 ± 0	29 ± 2	27 ± 1	43 ± 2	41 ± 1
	28 kHz	23 ± 1	27 ± 1	28 ± 1	28 ± 1	40 ± 2	39 ± 1
	40 kHz	28 ± 1	30 ± 2	32 ± 1	29 ± 1	39 ± 2	38 ± 1
Peak latencies^1^ (ms)	Wave I	1.42 ± 0.01	1.33 ± 0.02	1.29 ± 0.04	1.35 ± 0.02	1.39 ± 0.02	1.34 ± 0.04
	Wave II	2.42 ± 0.03	2.36 ± 0.02	2.18 ± 0.04	2.31 ± 0.02	2.36 ± 0.03	2.31 ± 0.07
	Wave III	3.17 ± 0.04	2.99 ± 0.05	2.87 ± 0.06	2.97 ± 0.03	3.17 ± 0.06	3.20 ± 0.09
	Wave IV	4.07 ± 0.03	3.93 ± 0.04	3.90 ± 0.05	3.93 ± 0.02	4.06 ± 0.03	3.99 ± 0.04
Interpeak latencies^1^ (ms)	I–II	1.00 ± 0.02	1.02 ± 0.01	0.98 ± 0.02	0.96 ± 0.01	0.97 ± 0.02	0.97 ± 0.03
	I–IV	2.65 ± 0.03	2.60 ± 0.03	2.61 ± 0.03	2.58 ± 0.02	2.68 ± 0.02	2.65 ± 0.03
	II–IV	1.65 ± 0.02	1.57 ± 0.02	1.72 ± 0.02	1.62 ± 0.02	1.70 ± 0.02	1.68 ± 0.05
Amplitude^1^ (nV)	Wave I	857 ± 88	729 ± 95	869 ± 217	835 ± 44	553 ± 39	424 ± 57
	Wave II	1474 ± 153	1285 ± 128	922 ± 147	1035 ± 73	961 ± 79	964 ± 90
	Wave III	150 ± 44	188 ± 52	278 ± 66	288 ± 65	192 ± 35	224 ± 60
	Wave IV	1278 ± 175	1105 ± 104	1227 ± 210	1449 ± 106	746 ± 94	797 ± 129

*Hearing parameters were measured during the whole study in the normal (NF) and folate deficient (FD) dietary groups, although only three key points of the experiment that correspond to the start, 5 and 8 months of diet administration (2, 7 and 10 months of age) are shown. ^1^mean ± SEM*.

The presence of the *2f1-f2* component after pure tone stimulation was evident in both groups in DPOAE studies. However, FD-10M mice presented increased DP thresholds at all the frequencies studied as compared to NF-10M mice, the differences being significant at 8–14 kHz (Figure 2B). Concomitantly, decreased *2f1-f2* amplitude was detected in the FD-10M group, which became significant at 10 kHz (Figure 2Bb). These results suggested that the initial signs of hearing loss and the outer hair cell damage were more pronounced in the FD than in the NF group after 8 months of nutritional intervention.

## Discussion

The relationship between nutritional deficiencies in vitamins, including folate deficiency, and hearing loss has been explored in a variety of epidemiological studies (Houston et al., 1999; Cadoni et al., 2004; Lasisi et al., 2010; Attias et al., 2012; Karli et al., 2013). However, the data obtained have not always been consistent. This fact, led several authors to the evaluation of the putative contribution of human mutations to these disparities and, among them, the analysis of modifications in key genes of the one-carbon metabolism. Evaluation of the relationship between the polymorphism C677T in the methylenetetrahydrofolate reductase gene, a key player in folate metabolism, and hearing loss rendered contradictory results (Durga et al., 2006; Uchida et al., 2011; Fusconi et al., 2012). In contrast, animal studies have provided clues to the interplay between folate, Hcy and hearing loss, showing alterations in Hcy metabolism correlating with the onset of this impairment (Cohen-Salmon et al., 2007; Kundu et al., 2012; Martinez-Vega et al., 2015a). In this line of evidence, a previous study using the C57BL/6J mouse strain, known by its early onset of hearing loss, showed that a decrease in folate intake accelerates development of this sensory decline (Martinez-Vega et al., 2015a). A fact that correlated with a systemic decrease of the vitamin levels and concomitant hyperhomocysteinemia, as expected from the critical role of folate in Hcy remethylation (Pajares and Pérez-Sala, 2006). These systemic changes are also detected in the present study using CBA/Ca mice during long-term feeding with a folate deprived diet, following the same trend than in C57BL/6J mice after 2 months of FD diet (Martinez-Vega et al., 2015a). However, hyperhomocysteinemia was more severe in CBA/Ca mice after 8 months on the FD diet than in C57BL/6J mice after 2 months (Martinez-Vega et al., 2015a).

Folate deficiency induced premature signs of hearing loss in both strains, although their appearance is delayed more than 6 months in the CBA/Ca strain as compared to C57BL/6J mice (Martinez-Vega et al., 2015a). Moreover, the extent of the damage is also very different, since most C57BL/6J mice showed profound hearing loss after 2 months on the FD diet (Martinez-Vega et al., 2015a), whereas the changes in hearing parameters in CBA/Ca mice are small, but consistent (increased DP thresholds and *2f1-f2* amplitude) and appear after 8 months on the deficient diet. In fact, histological data corroborated cochlear damage in CBA/Ca mice that was more evident in the FD animals. Folate-induced damage seemed restricted to the low basal turn of the cochlea, thus explaining that stimulation required high frequency sounds (Müller et al., 2005). This higher resistance of the CBA/Ca cochleae against vitamin deficiency may rely in differences in the genetic background of the strains used that remain largely unknown (Willott et al., 1995).

The high resistance against FD-induced hearing loss exhibited by CBA/Ca mice, as compared to the C57BL/6J strain used in our previous work (Martinez-Vega et al., 2015a), suggested putative differences in genes involved in folate metabolism. However, the small size of the mouse cochleae and the histological procedures carried out precluded an expression study on the remaining samples. Nevertheless, a search carried out into the Mouse Genome Project database showed a number of single nucleotide polymorphisms (SNPs) on several genes of folate metabolism in CBA/J mice as compared to the reference C57BL/6J strain (Keane et al., 2011). No differences in *Mtr*, *Bhmt*, *Dhfr*, *Tyms*, *Mthfd1* and *Mthfd2* genomic sequences were found between both strains (Table 3). In contrast, several SNPs for *Bhmt2*, *Folr1*, *Folr2*, *Mtrr*, *Mthfr*, *Shmt1* and *Shmt2* were identified. Among them, a missense mutation on *Folr1* for which the functional impact remains unknown, and those on *Bhmt2*, a gene whose hepatic expression levels have been proposed as a dietary-dependent factor conferring protection against drug intoxication (Liu et al., 2010). In this work, Liu et al. (2010) suggested that strains with higher hepatic BHMT2 levels/activity would be adapted for a lower dependence on folate for Hcy recycling by the use of S-methylmethionine as methyl donor. Clarification on whether this postulate is valid for the auditory system would require an extensive work on genomic sequencing and evaluation of several SNPs that is out of the scope of the present article.

**Table 3:** **Single nucleotide polymorphisms (SNPs) for key genes of folate metabolism detected in CBA/J mice according to the Mouse Genome Project**.

Gene	Base in C57BL/6J	SNP	Base in CBA/J	Main change
*Folr1*	G	rs47738978	A*	3’-UTR
	A	rs47631647	G*	missense F236L
	G	rs47445319	A*	upstream 2KB
	A	rs49747152	G*	upstream 2KB
	C	rs50854846	A	intron
	C	rs52411810	A	intron
	A	rs586270005	T	intron
	C	rs52321323	T	intron
	C	rs52448898	A*	upstream 2KB
	G	rs240085681	C	intron
	G	rs262701625	A	intron
*Folr2*	C	rs32160123	G*	intron
	C	rs31011801	T*	intron
	A	rs31275071	C*	intron
	T	rs48667177	A*	intron
*Bhmt2*	C	rs264842124	T	downstream 500B
	T	rs47046885	C	3’-UTR
*Mthfr*	A	148039163^a^	G*	unknown
	G	148039229^a^	C*
	C	148039557^a^	G
	C	148040119^a^	T
	C	148040240^a^	T
*Mtrr*	T	rs108123629	t/a	3’-UTR
	C	rs108647820	c/a	3’-UTR
	G	rs108535269	G	3’-UTR
	T	rs108864771	T	3’-UTR
*Shmt1*	A	rs26957199^b^	G*	3’-UTR
	A	rs47721266	C*	downstream 500B
	A	rs49213375	C*	downstream 500B
	T	rs222704054	C*	downstream 500B
	A	rs222753876	G*	downstream 500B
	A	rs108000000	G*	downstream 500B
	C	rs26957196	A*	downstream 500B
	G	rs45981582	A*	downstream 500B
	G	rs26957195	A*	downstream 500B
*Shmt2*	G	rs252755979^b^	T*	3’-UTR
	C	rs240177349^b^	T*	3’-UTR
	C	rs51081486^b^	T*	3’-UTR
	A	rs47587430	G*	3’-UTR
	C	rs260368105	T*	3’-UTR
	G	rs49954613	C*	3’-UTR

The table shows results of a search for SNPs in key genes of folate metabolism of CBA/J mice carried out in The Mouse Genome Project web page (http://www.sanger.ac.uk/science/data/mouse-genomes-project), where the C57BL76J strain is used as reference. *Multiple consequences, only the main one listed. ^a^Number indicates position. ^b^Gene assignment differs between MGP and NCBI databases.

Despite the interest of a prolonged study to get deeper insight the interplay between Nutrition and cochlear aging, the continuity of this long-term nutritional treatment was precluded by the presence of megaloblastic anemia. This fact was described in previous studies analyzing low intakes of folate or vitamin B_12_ for extended periods of time (Cox et al., 1961; Bills et al., 1992), but was not evident in C57BL/6J mice after 2 months of diet (Martinez-Vega et al., 2015a). Altogether, our data support the role of folate deficiency in premature development of hearing loss, although new experiments aimed to analyze its impact in strains with delayed onset of this sensory impairment should consider beginning nutritional interventions at later stages of animal development. Moreover, studies on the putative value of folate supplementation against hearing loss are needed, as well as the analysis of the incidence of this sensory decline in human populations living in countries with mandatory folate fortification for which only preliminary data are available: http://www.cdc.gov/ncbddd/hearingloss/data.html. In this line, nutrigenomic studies may render clues about the interaction between folate deficiency and the cochlear genome.

## Conclusion

The results presented herein reinforce the crucial role of folic acid status in the cochlea. Moreover, our data suggest that differences encountered in animal and human studies analyzing the interplay between nutritional deficiencies and hearing loss, especially in age-related hearing loss, may depend on the combinatory effects of both diet and genetic background.

## Author Contributions

RM-V carried out animal experimentation, histological evaluation and statistical analyses; RM-V and SM-C performed hearing assessment; TP and GV-M performed metabolite analysis; IV-N, GV-M and MAP conceived, designed and coordinated the study; MAP drafted the manuscript. All authors gave final approval for publication.

## Funding

RM-V was a fellow of the JAE-CSIC predoctoral program. This work was supported by grants of the Ministerio de Economía y Competitividad (SAF2014-53979-R to IV-N; BFU2009-08977 to MAP), the European Union (FP7-AFHELO and TARGEAR to IV-N).

## Conflict of Interest Statement

The authors declare that the research was conducted in the absence of any commercial or financial relationships that could be construed as a potential conflict of interest.

## Footnotes

^1^http://www.envigo.com/resources/growth-curves/growth-curve-cbacaola_505_b2_2013.pdf
